# The Use of Live Cell Imaging and Automated Image Analysis to Assist With Determining Optimal Parameters for Angiogenic Assay *in vitro*

**DOI:** 10.3389/fcell.2019.00045

**Published:** 2019-04-10

**Authors:** Brooke M. Huuskes, Ryan J. DeBuque, Peter G. Kerr, Chrishan S. Samuel, Sharon D. Ricardo

**Affiliations:** ^1^Department of Anatomy and Developmental Biology, Biomedicine Discovery Institute, Monash University, Melbourne, VIC, Australia; ^2^Australian Regenerative Medicine Institute, Monash University, Melbourne, VIC, Australia; ^3^Department of Nephrology, Monash Medical Centre, Monash University, Melbourne, VIC, Australia; ^4^Department of Pharmacology, Biomedicine Discovery Institute, Monash University, Melbourne, VIC, Australia

**Keywords:** endothelial progenitor cell, image analysis, angiogenesis, optimization, kidney disease

## Abstract

Testing angiogenic potential and function of cells in culture is important for the understanding of the mechanisms that can modulate angiogenesis, especially when discovering novel anti- or pro-angiogenic therapeutics. Commonly used angiogenic assays include tube formation, proliferation, migration, and wound healing, and although well-characterized, it is important that methodology is standardized and reproducible. Human endothelial progenitor cells (EPCs) are critical for post-natal vascular homeostasis and can be isolated from human peripheral blood. Endothelial colony forming cells (ECFCs) are a subset of EPCs and are of interest as a possible therapeutic target for hypoxic diseases such as kidney disease, as they have a high angiogenic potential. However, once ECFCs are identified in culture, the exact timing of passaging has not been well-described and the optimal conditions to perform angiogenic assays such as seeding density, growth media (GM) concentrations and end-points of these assays is widely varied in the literature. Here, we describe the process of isolating, culturing and passaging ECFCs from patients with end-stage renal disease (ESRD), aided by image analysis. We further describe optimal conditions, for human bladder endothelial cells (hBECs), challenged in angiogenic assays and confirm that cell density is a limiting factor in accurately detecting angiogenic parameters. Furthermore, we show that GM along is enough to alter the angiogenic potential of cells, seeded at the same density. Lastly, we report on the success of human ECFCs in angiogenic assays and describe the benefits of live-cell imaging combined with time-lapse microscopy for this type of investigation.

## Introduction

Angiogenic assays are commonly used to determine the ability of specific compounds to promote or inhibit angiogenesis. In kidney disease, the discovery of agents that can restore microvascular circulation after injury is highly sought after, as the common end point of all kidney injury is fibrosis resulting in decreased oxygen perfusion and blood flow loss to the kidney. Whilst angiogenesis is stimulated in the early stages of kidney disease, there is a rapid switch to a hypoxic state which denudes the angiogenic processes (Long et al., 2012). It is well-documented that the decline of endothelial progenitor cells (EPCs) in the circulation is linked to poor outcomes for patients with chronic kidney disease (Povsic and Goldschmidt-Clermont, 2008; Goligorsky et al., 2010). Since their discovery in 1997 (Asahara et al., 1997), EPCs have been suggested as a potential therapeutic target for a range of vascular disorders, yet translation to clinical therapies has shown limited success due to difficulties in the expansion of EPCs into appropriate numbers due to senescence of the isolated cells (Chong et al., 2016). Historically, the source of EPCs has been from large volumes of easily accessible blood, such as cord blood and more recently, homogenates of specific organs in animal models (Mead et al., 2008; Alphonse et al., 2015). However, personalized medicine is growing in popularity due to the immunological issues that arise from allogenic transplants, hence autologous therapy is widely being investigated. In going forward it is important to have consensus on the methodology used to isolate and culture cells to be used as therapies in patients.

Testing the function of EPCs requires their maturation to a endothelial colony forming cell (ECFC), which can be achieved via an established culture method (Mead et al., 2008). ECFCs are comparable to mature endothelial cells and can act in similar ways when challenged in functional assays as they have increased angiogenic potential over EPCs (Ingram, 2004; Hirschi et al., 2008). The transition from an EPC to an ECFC cell can be first identified by a change in morphology, which requires daily investigation by microscopy. Care needs to be taken when conducting these experiments as fluctuations in temperature and oxygen levels can alter experimental outcomes (Francescone et al., 2011). The exact timing of when to passage the ECFC colonies has not been consistently reported, varying from suggested times of 14 days (Mead et al., 2008) or when colony size reaches 1,000 cells (Zhao et al., 2014), yet it is documented that ECFCs appear at various times depending on disease setting and the source of EPCs (Choi et al., 2004; Zhao et al., 2014; Gu et al., 2015). These important parameters are addressed in the current study that describes an image based, quantitative method to determine the optimal timing for ECFC passaging from small starting volumes of blood.

Investigation of ECFC function can be measured by assessing proliferation capacity, invasion through basement membranes and most commonly, *in vitro* tube formation of which tube length is the main parameter recorded (Staton et al., 2009). However, there has been no consensus on optimal conditions for cell isolation and culture, such as seeding density, growth media (GM) concentrations and end points, with these experimental procedures in the literature significantly varied. These considerations are critical when planning angiogenic experiments as they can alter the angiogenic response and without consensus across the field of endothelial biology there is limited ability to make meaningful comparisons in the literature. We demonstrate the optimal culture conditions of human bladder endothelial cells (hBECs) by titrating both the number of cells and GM concentration in tube forming assays. We then use this data to characterize the function of ECFCs derived from dialysis-dependent kidney disease patients. This cohort of patients was chosen to develop a protocol where ECFCs were isolated and propagated from small volumes of blood in a chronic inflammatory condition regarded as a common comorbid condition in ESRD and especially in dialysis patients. Further, we describe in detail the benefits of using live cell imaging using these various cell lines in a custom-built incubation chamber using time-lapse microscopy. We further describe how image analysis and custom designed macros programmed with the language of FIJI are used to streamline manual processes. Together, the results described in this manuscript will enable accurate, reproducible results and provides a consensus regarding the appropriate method to conduct and analyze the angiogenic response of isolated cells.

## Materials and Methods

### Human Samples

All human studies were approved by the Monash Health Human Research Ethics Committee (CF16/402 - 2016000182), which conforms to the National Statement on *Ethical Conduct in Human Research*. Patients with end-stage renal disease (ESRD) who were dialysis dependent were recruited from Monash Medical Centre [Melbourne, Australia (*n* = 20)] and participated in this study under informed consent. Patients were excluded from the study if their original diagnosis of ESRD was type I/II diabetes, or who were on antibiotics or had a recent infection or inflammatory flare-up.

### Blood Sampling, Isolation, and Culture of EPCs

Blood sampling was conducted as previously described (Huuskes et al., 2018). Briefly, blood (10 mL) was collected in VACUETTE Premium K2E K2EDTA tubes (Greiner bio-one, Kremsmunster, Austria) from patients prior to a single dialysis session. Blood was diluted and separated based on density using Ficoll (GE Healthcare Life Sciences, Uppsala, Sweden). The peripheral blood mononuclear cell (PBMC) fraction was plated in 6-well culture dishes (2.5 × 10^6^/well) coated with human fibronectin (2 μg/cm^2^ plated at minimal volume) and maintained in Endothelial Growth Medium (EGM-2, cat# CC-3202, Lonza, Mount Waverly, Australia) as previously described (Kalka et al., 2000). Nonadherent cells were removed 3 days after culture. Media was changed every second day until ECFCs were harvested, or to a maximum of 30 days.

### ECFC Identification

Plates containing PMBC fractions were inspected via daily microscopy (LX AF6000, Leica Microsystems, North Ryde, Australia) to determine if an ECFC colony had differentiated. This was determined by a change in morphology and rapid proliferation of cells and occurred anywhere between 7 and 10 days (Huuskes et al., 2018). ECFC colonies were passaged only when the cell density reached ~2,000 cells. To determine this, images of individual colonies were captured using the LX microscope (Leica) every day once the initial colony was seen and cell number was analyzed using macro written in the language of FIJI (Schindelin et al., 2012) (FIJI 2.0.0-rc-49/1.51s, National Institute of Health, available for download: www.imagej.net). Once a mature phenotype was reached colonies were passaged as previously described (Zhao et al., 2014). Briefly, colonies were incubated with trypsin (0.05%, Lonza) and loosened by pipetting media directly over the colony. Up to 3 mLs of EGM-2 was used to collect a single colony and added to a fibronectin coated T-25 flask. Cells were incubated and media was changed every second day until flask reached 80% confluence.

### Optimization of *in vitro* Tube Forming Assay

Studies were conducted with commercially purchased primary bladder endothelial cells (Lonza, Cat# CC-7016) and used in all assays to determine the optimal number of cells and growth media concentration in tube forming assays. These cells were shipped at passage 3 and were negative for viruses and microbes with a doubling time of ~20 h and a 20% seeding efficiency.

### Tube Formation on Matrigel

Tube formation was conducted on Matrigel (Corning MA, USA Cat# 356231) in 96-well-plates (BD Falcon) as previously described (Gu et al., 2015) with the following adjustments. Cells were plated at decreasing cell density of 10 × 10^4^, 7.5 × 10^4^, 5 × 10^4^, 2 × 10^4^, 0.5 × 10^4^, and 0.2 × 10^4^ in triplicate in 100 μL of 100% GM. Time-lapse microscopy was utilized on the LX AF6000 microscope (Leica) imaging the cells every 5 min for 6 h. The microscope was enclosed by a custom made perspex housing and contained a custom made lid (both made by www.clearstatesolutions.com, Figure S1) allowing the microscope to operate in an incubator for live cell imaging over extended periods of time. The whole perspex chamber and stage of the microscope were heated to 37°C with the lid connected to a CO_2_ line which bubbled into a reservoir containing milliQ water. This allowed the cells to be incubated in a temperature controlled environment with 5% CO_2_ in a humidified chamber.

Additional experiments were conducted in order to determine the optimal GM concentration, which allowed cells to show either an increase or decrease in tube formation ability based on the angiogenic stimulating or inhibiting factors added to the culture media. This was performed as described above with 0.5 × 10^4^ cells per well and GM was titrated at; 100, 75, 50, 25, 10, 1, and 0% where by 100% GM was supplemented with EBM-2 media (basal medium, Lonza, Cat# CC-3156).

In order to determine if time-lapse microscopy could capture cell-to-cell interactions using multiple channels over extended time periods, hBECs were placed in tube formation assays with mesenchymal stem cells (MSCs) expressing the enhanced green fluorescent protein (eGFP). MSCs were purchased from the Tulane Centre for Stem Cell Research and Regenerative Medicine (Tulane University, New Orleans, LA, USA) and cultured as described previously (Huuskes et al., 2015). MSCs were phenotypically and karyotypically normal and expressed the GFP (Wise et al., 2014). For consistency, the number of cells per well in the hBEC-MSC co-culture experiment was kept at 0.5 × 10^4^, where MSCs were plated with the hBECs at a 1:4 ratio. A macro was written in the langue of FIJI to allow images at specific timepoints to be merged from multiple channels and saved over the whole 7 h experiment. For consistency, the number of cells per well in the hBEC-MSC co-culture experiment was kept at 0.5 × 10^4^, where MSCs were plated with the hBECs at a 1:4 ratio.

### Time-Lapse Microscopy

Using the LX AF6000 microscope and the Leica Application Suite software (Leica) cells were imaged at regular intervals over an extended period of time (The Mark and Find Figure S2D). function allowed the center of the wells to be defined and saved, and recalled with high precision accuracy to allow images of the same position to be imaged over time. Care was taken to ensure that no air bubbles were present in the Matrigel or the media as this interfered with analysis (Figure S2E). Images were captured with a 5x objective (Leica, HC PL FLUOTAR, dry immersion lens, aperture 0.15, working distance 12,000) positioned in the center of the well and imaged every 2 min 30 s over 6–7 h or every 5 min beyond 6 h. Images were opened as a group in FIJI ^17^ and exported as an .AVI movie at a frame rate that would result in a video of ~4–10 s to allow visualization of tube forming kinetics.

For the hBEC-MSC co-culture experiment, the microscope was set up as above with minor adjustments. A second channel containing the 488 laser was activated and images were captured sequentially in a bright field-FITC manner. The resulting file when opened for analysis displayed all bright field images stacked, and all fluorescent images stacked separately.

### Analysis Macro

In order to determine the optimal time and GM concentration for tube formation assays, a macro programmed with the language of FIJI was developed to automatically identify images at every hour and save. The images were subsequently analyzed with the angiogenesis plug in Carpentier et al. (2012) for FIJI software (Schindelin et al., 2012), which automatically identified and analyzed 20 angiogenic parameters, of which tube length, number of junctions, number of branches and number of meshes were reported. Determining the interaction between hBECs and MSCs was streamlined, programmed with the macro language of FIJI, written to merge bright field and fluorescent images.

### Human ECFCs

ECFCs derived from dialysis patients were also challenged in the above angiogenic assays after the optimal cell seeding density (0.5 × 10^4^) and growth media concentration (25%) were determined with hBECS. ECFCs were characterized as mature endothelial cells as according to their cell surface markers by both flow cytometry (Huuskes et al., 2018) and immunocytochemistry as previously described (Herbrig et al., 2004). Briefly, patient derived ECFCs were subjected to flow cytometry after staining with single colors or an antibody cocktail for mouse anti-human CD31 APC (1:400), mouse anti-human CD34 PE-Cy7 (1:50), anti-KDR PE (1:20), anti-CD45 BV510 (1:200, all BD Bioscience). Appropriate isotype controls were also used at the same concentration as corresponding test antibodies for each experiment. Additionally, ECFCs were cultured on glass slides and incubated 1 μg/mL of FITC-labeled Ulex Europaeus Lectin (Sigma) and 10 μg/mL of Dil-ac-LDL (Thermo Scientific) for 3 h at 37°C. Additionally, cells were stained with primary rabbit antibodies against CD31 (1:100, cat # ab28364, abcam), von Willebrand (vWF) (1:100, cat # A0082, Dako) and VEGFR2 (1:100 cat # ab2349, abcam). Rabbit serum was used as an isotype control and all sections were incubated overnight at 4°C. Wells were washed in PBS and incubated with anti-rabbit 555 secondary antibody for 1 h at 37°C and counterstained with DAPI. All ECFCs were visualized using laser confocal microscopy (Leica SP8 HyD Confocal Invert).

Human derived ECFCs (*n* = 3 patients, in technical triplicate) were also challenged in tube forming assays using the optimal conditions as determined using hBECs. Patent ECFCs were therefore seeded density of 0.5 × 10^4^ cells per well in a 96 well-plate with 25%GM with or without the addition of MSCs. Time-lapse microscopy was used as described above to capture images, which were analyzed using the macro describe above.

### Statistical Analysis

All data is expressed as mean ± SEM and was analyzed using GraphPad Prism. For correlation analysis, a Pearson correlation was conducted for normally distributed data, and a Spearman nonparametic correlation was conducted when data did not follow a normal distribution. For comparison of multiple groups a one-way ANOVA was performed with a Tukey's *post-hoc* test. A *p*-value of < 0.05 was considered statistically significant.

## Results

### ECFCs Are Passaged Once Colony Density Reaches ~2,000 Cells

**Figure 1:**
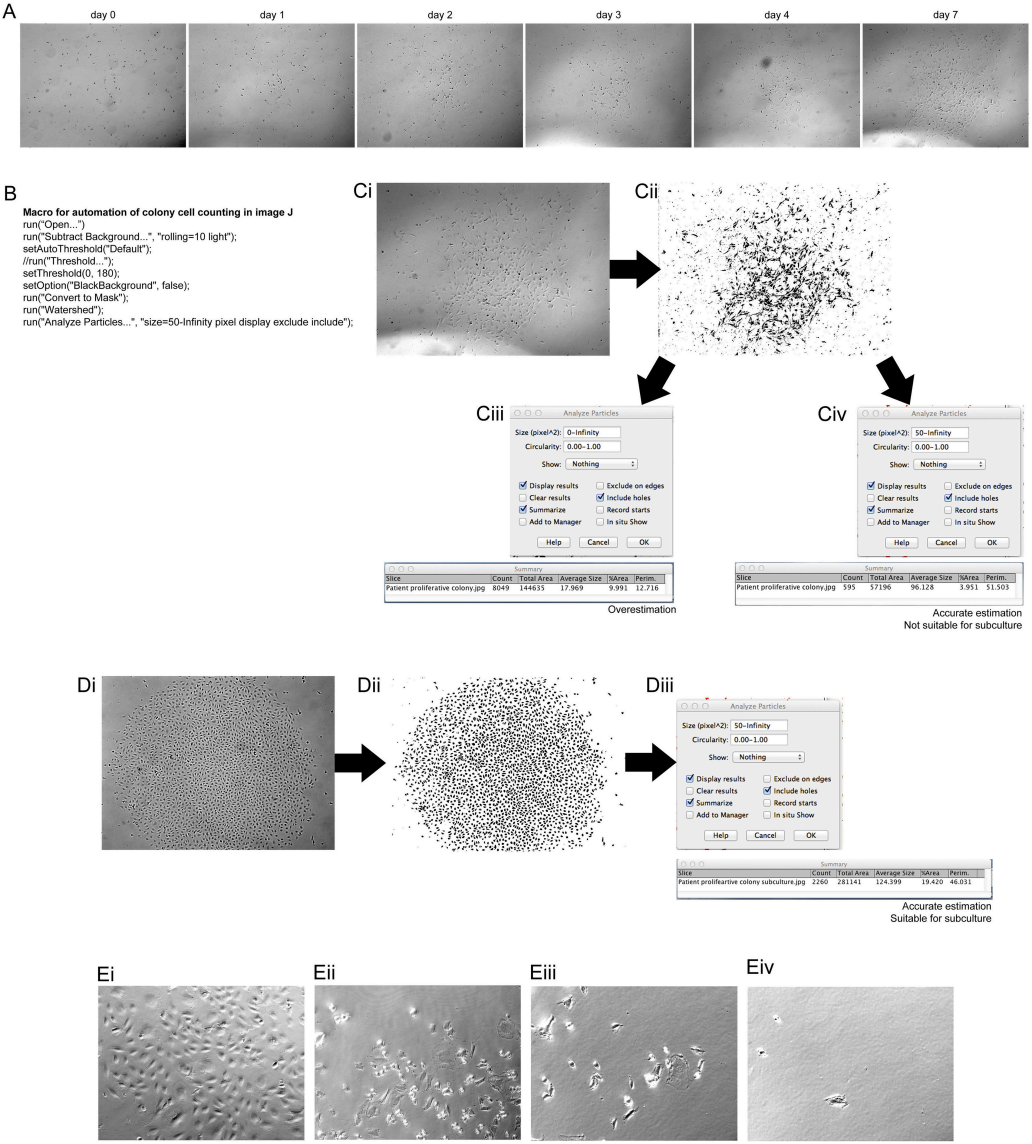
Assessment of clonal expansion of patient isolated EPCs and the use of image J to inspect colony size. Morphological changes of patient isolated PBMCs at 7 days of initial culture showed transformation into a mature, proliferative colony. **(A)** Patient EPCs transformed into a proliferative ECFC colony. The use of the “mark and find” function on the Leica software allowed the colony growth to be monitored over consecutive days. At day 7 the colony was imaged and assessed using a macro, programmed with the macro language of FIJI **(B)** to determine if the colony was the correct size (>1,000 cells) for passaging. The image of the colony was transformed from a bright field image **(Ci)** to a binary image **(Cii)** using the FIJI macro. When analyzing the particles in the image, it is important to choose the correct size of the pixels to be analyzed. If the pixel size is too small **(Ciii)** then an overestimation of the number of cells can result. We found that a pixel size of 50-infinity **(Civ)** provided the most accurate representation of the number of cells present. For this representative patient, this method confirmed that the colony size was not sufficient to passage, and therefore a continued culture time was required. An example of a patient colony that was ready to passage **(Di)**, after transforming to a binary image, **(Dii)** using the FIJI macro. A total of 2,260 cells was observed **(Diii)**, therefore the colony was passaged as confirmed by using the FIJI macro. A total count of 2,260 cells was observed, therefore colony was passaged. Proliferative colonies can be detached without cylinders by incubating with a dissociation agent and forcefully pipetting media over the colony over a minimum of two washes Proliferative colonies can be detached without cylinders **(Ei)** by incubating with a dissociation agent and forcefully pipetting media over the colony **(Eii)**. After one wash **(Eiii)** there are still some cells attached, however using two washes removes the majority of the cells **(Eiv)**. **(E)** Scale Bars A–D: all 500 μm. Scale bar E: 250 μm.

For expansion of ECFCs into appropriate numbers for investigation of angiogenic potential it was important to first determine if the number of cells in the colony were appropriate for harvesting. PBMCs were investigated on the LX AF6000 every day from 7 days post PBMC plating. Once proliferative colonies were seen, as determined by their change in morphology, they were imaged every day (Figure 1A). In order to determine if the colony size was sufficient for passaging, a macro in FIJI (Schindelin et al., 2012) was programmed (Figure 1B) to rapidly analyze cell number. The colony of interest (Figure 1Ci) underwent processing whereby; (1) the background was removed from the image; (2) a threshold was set in order to mark all the cells to be counted; and (3) was converted to a binary image (Figure 1Cii). The last step in the macro was to “analyze particles” and the size in pixels^∧^2 as it was very important to determine the correct cell number, especially as the images was not very clear and had other cells and debris included. An over representation of cell number can easily be determined (Figure 1Ciii) if the pixel size to be analyzed was zero-infinity, however if the pixel size was set as 50-infinity the correct cell number can be determined (Figure 1Civ). The colony analyzed however, did not have sufficient cell numbers (>1,000 cells) to be passaged. A second colony (Figure 1Di) was imaged and the macro was applied creating a binary image (Figure 1Dii). In the “analyze particles” dialog box, a pixel size of 50-infinity was chosen and the correct cell number was determined, indicating that the colony was ready for passaging (Figure 1Diii).

Previously studies using EPCs lack experimental detail on the method used to harvest ECFC colonies. Cylinder tubes with trypsin can be used^6^, however in this method no tubes were required. A colony was identified as being appropriate for harvesting and visualized (Figure 1Ei). After trypsin neutralization the cells started to detach from the well (Figure 1Eii), which was aided by the forceful washes using media (Figure 1Eiii). After 2 washes (Figure 1Eiv) 99% of the colony was removed from the well and was ready for passage 1 onto a fibronectin coated culture flask.

**Figure 2:**
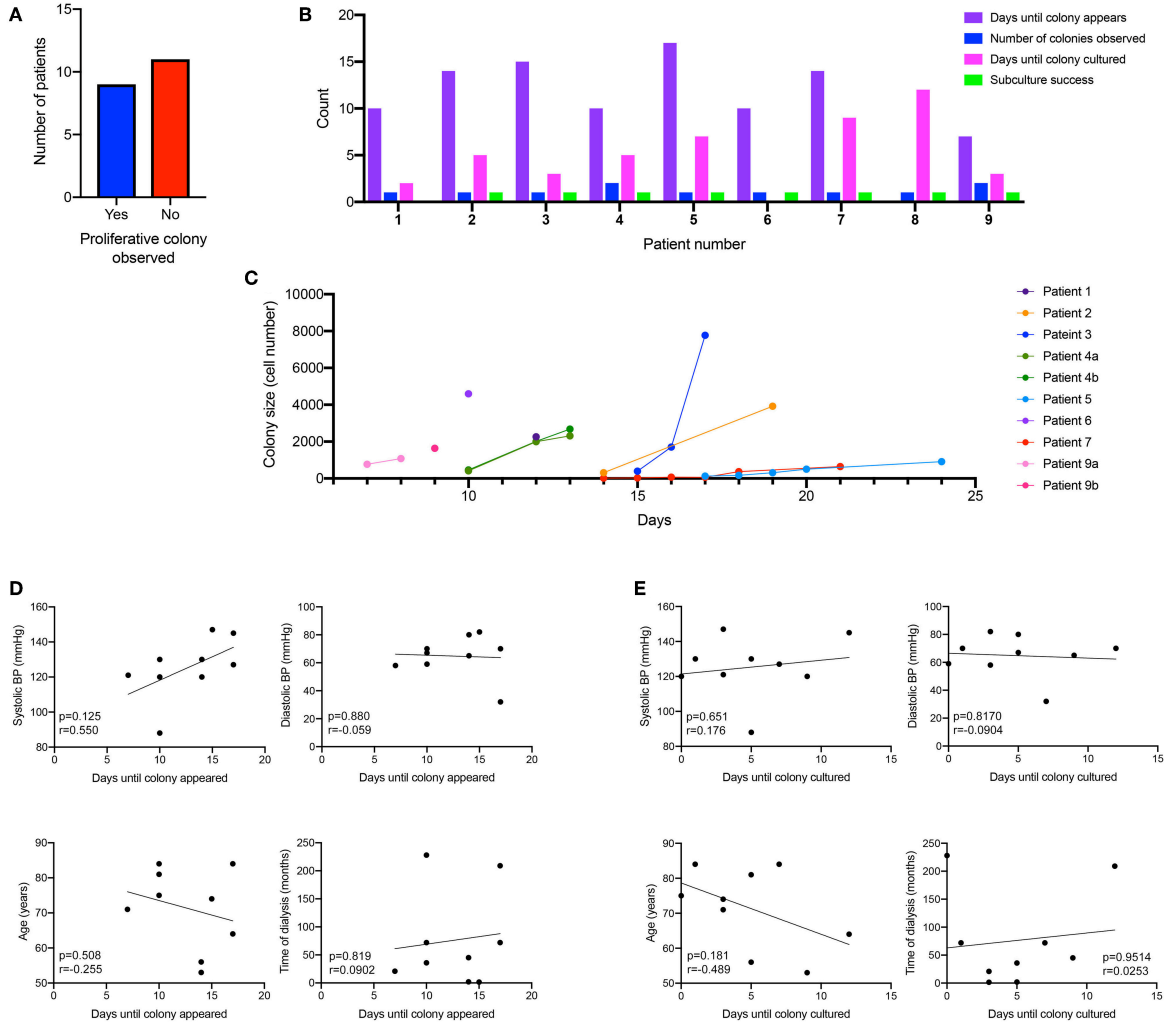
Kinetics of patient-derived proliferative ECFC colonies. Nine out of the 20 patient-cultured PBMCs went on to form a proliferative colony in culture **(A)**. Cells were cultured for 7 days and then visualized under a microscope to identify the morphological changes that occurred when ECFC colonies started to emerge. Patient-derived colonies first appeared in culture between 7 and 17 days **(B)** with some patients deriving multiple colonies. Subculture success was observed in 90% of the patient-derived cells that formed an ECFC. Colonies were monitored for their size and the number of days for the colonies to reach over 2,000 cells **(C)**. There was no correlation between blood pressure (systolic and diastolic), age or time on dialysis to when the ECFC colony first appeared **(D)** or when the ECFC colony reached a large enough size to culture **(E)**.

Using the macro, we could then determine the rate at which the ECFC colony formed. We did observe that the culture time of patient PBMCs to yield and ECFC varied, with some ECFCs appearing as early as 7 days after initial plating, others taking up to 17days for the ECFC to first appear. 45% of patient PBMCs gave rise for an ECFC colony over 2000 cells that were harvested for expansion (Figure 2A), which took an average of 5 days once the first change in morphology was observed. Of those, 78% of patient PBMCs yielded one ECFC, and 22% yielded two ECFC colonies. 90% of ECFCs were successfully passaged once (Figure 2B). The time taken for patient derived ECFCs to reach a specific cell density varied between patients (Figure 2C). Some colonies grew to 2,000 cells overnight as the first sign of a change in morphology was observed when the colony density was already >2,000 cells (patients 1, 4, 6). Other colonies were slow growing (patients 5, 7) and took up to 17 days to reach colony densities of 2,000 cells. In order to determine if patient characteristics were influencing the time for either a colony to appear in culture (Figure 2D) or the time taken for that culture to mature for subculture (Figure 2E) a correlation analysis was conducted and it was found that blood pressure, age and time on dialysis had no influence on the culture on patient-derived ECFCs.

### Optimal Cell Number and Growth Media Concentration for Tube Forming Assay

**Figure 3:**
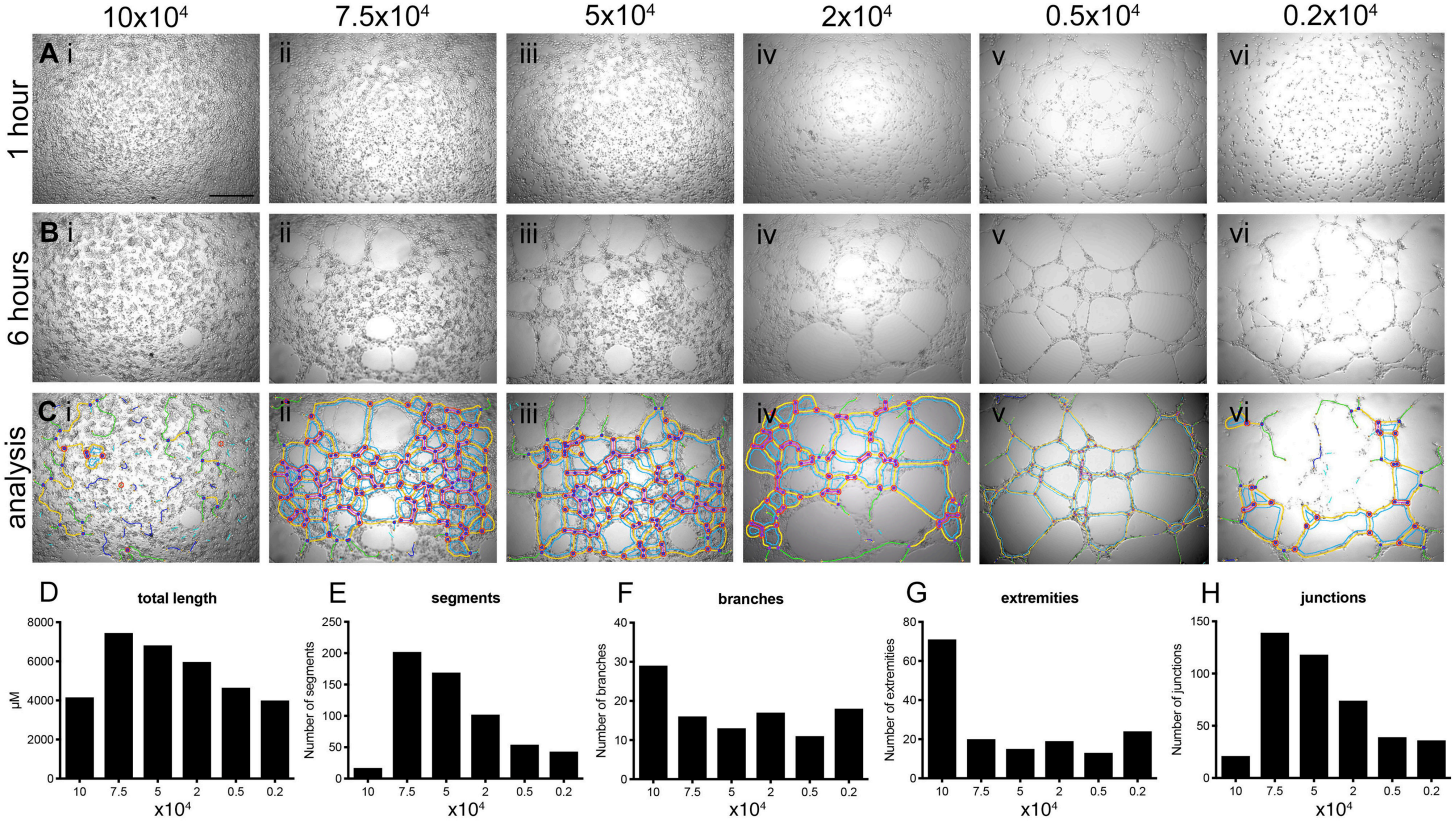
Determination of optimal number of cells for tube forming assay. hBECs were seeded at various densities in 96 well-plates coated with Matrigel and imaged using time-lapse microscopy over 6 h (top panels) and showing data analysis in graphs (lower panels). Images of tube formation were taken at 1 h **[A (Ai-Avi)]** and 6 h **[B (Bi-Bvi)]**. The images captured at 6 h were used to perform analysis with the angiogenesis plug-in for FIJI and the results of the analysis were overlaid with the bright field images **[C (Ci-Cvi)]**. Five parameters of the angiogenesis analyzer were reported as a measurement of tube forming ability; tube length **(D)**, the number of segments **(E)**, the number of branches **(F)**, the number of extremities **(G)** and the number of junctions **(H)** (*n* = 3, in triplicate). Scale bar: 500 μm.

hBECs were plated at various densities and cultured in 96-well-plates on Matrigel for 6 h. Live cell imaging was used to capture tube formation and representative images were exported as a time-lapse movie (Videos S1–S6). Cell densities over 2 × 10^4^ cells produced results that were unusable for determining angiogenic potential (Figures 3Ai–vi). This was confirmed by visualization of the skeletonized tree that was created by the angiogenesis analyzer and overlaid on the phase contrast images after 6 h of culture (Figures 3Bi–vi). Visualization of these trees (Figures 3Ci–iv) is required when determining the success of analysis, as there is a clear over-representation of parameters when a cell number of >2 × 10^4^ were used. This over-representation may not be as obvious when investigating the numerical output, such as total branch length (Figure 3D), number of segments (Figure 3E), number of branches (Figure 3F), number of extremities (Figure 3G) and number of junctions (Figure 3H), which are all common parameters used to determine the angiogenic potential of cells.

Based on these results all future experiments were conducted with 0.5 × 10^4^ cells (Figures 3Av, Bv, Cv). Importantly, successful tube analysis relies heavily on the quality of the image captured from the microscope, exemplified when images captured tube forming ability of 0.2 × 10^4^ cells (Figure 3Avi) where the center of the well is over exposed and affects the ability of the angiogenesis plug-in to correctly identify tube parameters (Figures 3Biv, Civ). The optimal microscope settings used for these studies are provided in Figures S2A–Cii.

**Figure 4:**
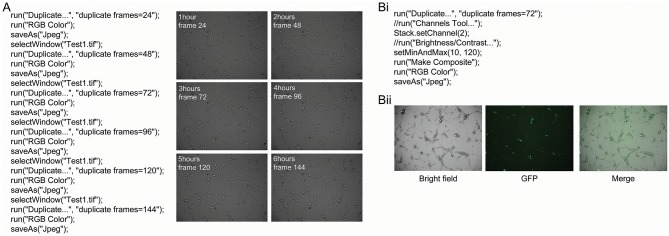
Macro to identify and save images from a series and merge bright field and fluorescent images. A macro was programmed with the language of FIJI to automatically identify images of interest from a time-lapse series **(A)**. This macro requires the time interval between images to be known so that specific time points can be identified and saved. In this example, images were captured every 2 min and 30 s for 6 h. Examples of the images that were obtained from this macro are shown, and had the angiogenesis plugin applied to analyze different parameters of tube formation. The AF LX6000 microscope can also take fluorescent images **(Bi)**. The relative focus control (RFC) function within the software allows two channels to be independently focused allowing both bright field and fluorescent images to be in focus simultaneously. If bright field and fluorescent images are taken together in a time-lapse they appear as two separate channels when opened in FIJI. A macro was programmed in order to identify the image of interest and the merge the bright field and fluorescent images **(Bii)** so that the interaction between two different cell types can be investigated.

Tube formation was imaged every 2 min and 30 s for 6 h when 0.5 × 10^4^ hBECs were plated in 96-well-plates on Matrigel and incubated on the LX AF6000 microscope. The media concentration was titrated from 100 to 0% in order to determine the GM concentration to assess both the increases and decreases that pro- or anti-angiogenic agents may have on tube formation Figure S3). A macro was written to quickly identify images from desired timepoints (Figure 4A). A second macro was written (Figure 4Bi) to demonstrate the ability of the microscope to analyze two channels. During time-lapse microscopy, images were taken sequentially from different channels and therefore appeared as two separate images when opened in FIJI. Therefore, the macro programmed identified the two sequential images and merged them (Figure 4Bii) so that the interaction between two different cell types could be investigated.

**Figure 5:**
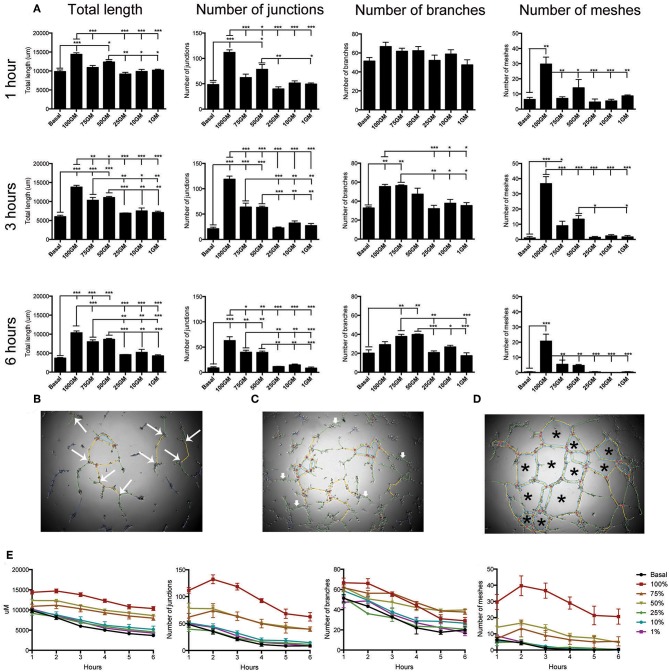
Quantification of tube angiogenic parameters over a 6 h tube forming experiment on Matrigel. Total tube length and the number of junctions, branches and meshes were analyzed over the 6 h of the tube formation experiment where hBECs were seeded at 5,000 cells/well on Matrigel in a 96 well-plate **(A)**. All parameters were significantly increased with the use of 100% GM as compared to basal media at all time points, with the exception of the number of branches at 1 h and 6 h. Maximal significant between all parameters and GM concentrations occurred at 3 h. The parameters that are included in this analysis were visualized as the number of junctions (large arrows, **B**). Branches did not make connections with any other tube (small arrows, **C**). Meshes are comprised of the complete joining of multiple segments and junctions (asterisk, **D**) Regardless of culture medium concentration, tube length, number of junctions, number of branches and number of meshes significantly decreased over the 6 h of the experiment **(E)**. *p* < 0.05 1 h vs. 6 h all growth medium concentrations (*n* = 3, in triplicate). GM, growth media, *p < 0.05, **p < 0.01, ***p < 0.001. Scale bar: 500 μm.

Representative images and subsequent quantification of these images using angiogenesis plug-in confirmed that 100%GM significantly increased tube length, and number of, junctions, meshes at all time points analyzed compared to basal media (Figure 5A, *p* < 0.001). The number of junctions (Figure 5B) also varied for the first 3 h, after which maximum significance was reached, and this difference between the top 3 media concentrations and the lower 3 became significant (*p* < 0.05) and obvious. In a similar fashion, the number of branches (Figure 5C) reached maximum significance at 3 h of culture between GM concentrations. Interestingly, after 4 h of culture the significance between basal media and 100% GM disappeared, likely indicating that the cells were starting to retract from each other and not participating their full branching potential. The most striking difference observed between GM concentrations was their effect on the number of meshes (Figure 5D) formed. Again, 100% GM had the most significant effect on the number of meshes, with more mesh formation at all time points analyzed, compared to all other GM concentrations (*p* < 0.05). At 3 h 50% GM did have significantly more mesh formation compared to 25% (*p* < 0.05) and 1% (*p* < 0.05) GM however this was reversed at all other subsequent time points Figure S4).

Results from the analysis of tube formation over 6 h also demonstrated that tubes had a maximal branching time, after which they start to retract away from each other (Figure 5E). This was determined by investigating total tube length, number of junctions, number of branches and number of meshes for each GM concentration over the total time of the experiment. These data clearly indicate that regardless of the concentration of GM used, this phenomenon of reduction in tube integrity was correlated with the length of assay time (*p* < 0.001 for each media concentration comparing parameter measured between 1 and 6 h).

### Human Derived ECFCs Characteristics

**Figure 6:**
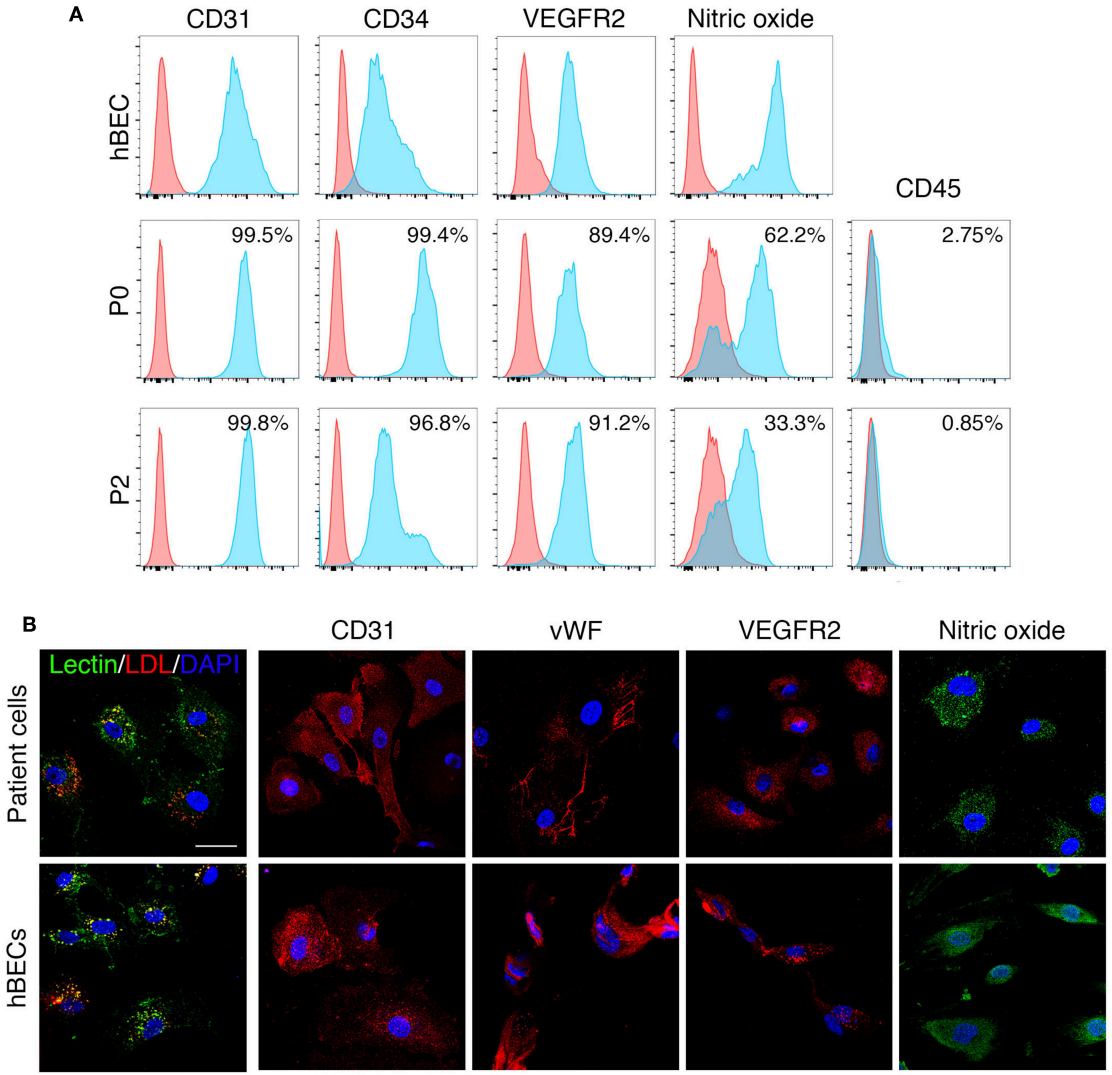
ECFC characteristics. ECFCs were isolated and cultured from peripheral blood collected from dialysis-dependent ESRD patients and were confirmed as mature endothelial cells via flow cytometry **(A)** and expression of specific proteins as determined by immunohistochemistry **(B)**.

Human derived ECFCs were confirmed as mature endothelial cells through the localization of specific cell surface proteins by both flow cytometry and immunohistochemical investigation. Patient cells maintained a mature endothelial cell phenotype at passage 2 (Figure 6A) as demonstrated by the presence of CD31, CD34, VEGFR2, and NO and absence of CD45 by flow cytometry. Similar to hBECs, patient derived cells stained positive for lectin and were able to engulf LDL and were positive for CD31, vWF, VEGFR2, and NO (Figure 6B).

**Figure 7:**
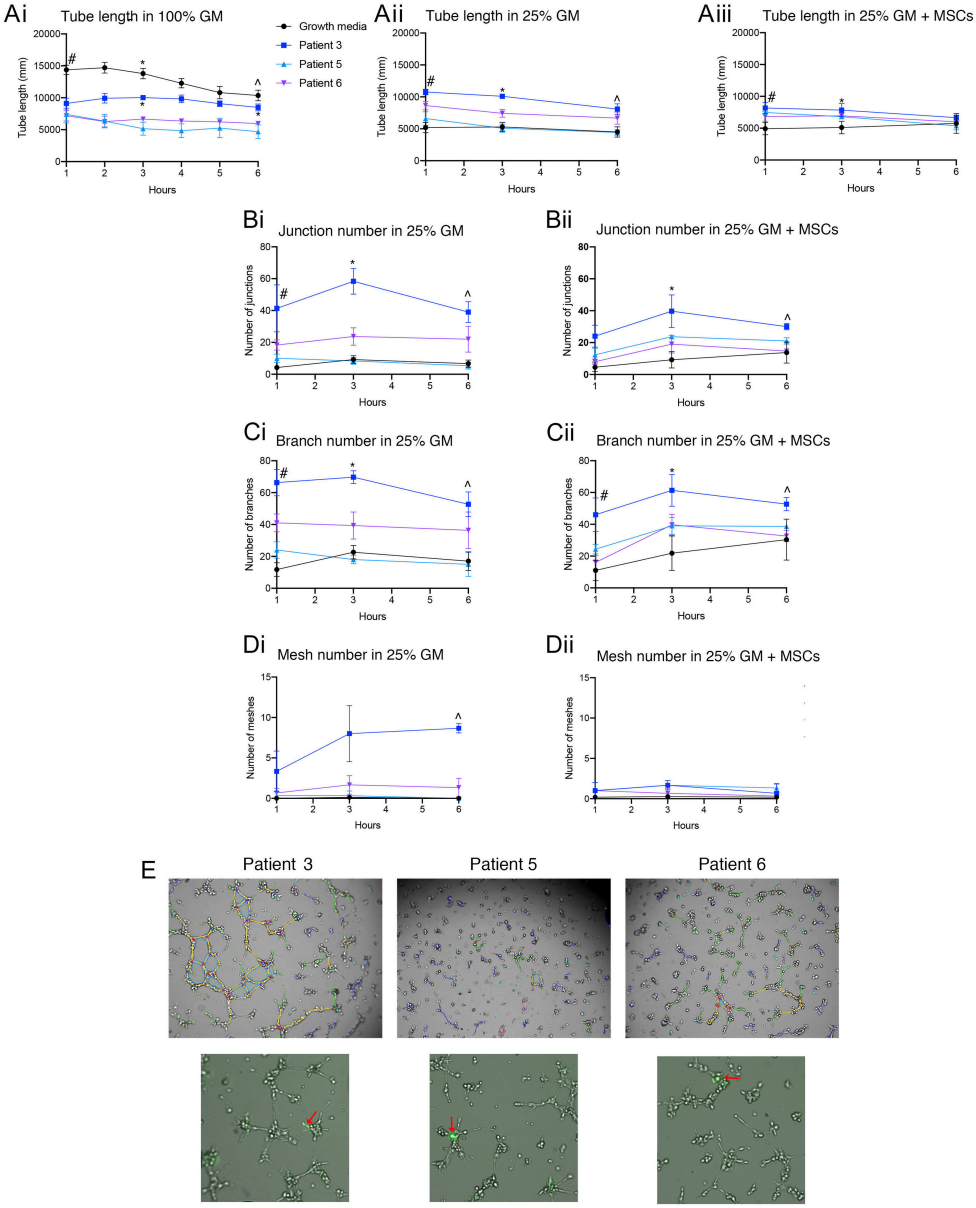
Quantification of patient-derived ECFCs angiogenic parameters over a 6 h tube forming experiment on Matrigel. Tube length was measured over 6 h in 100% GM, 25% GM, and 25% GM substituted with MSCs in both hBECs and patient-derived ECFCs **(A)**. Junction number **(B)**, branch number **(C)** and mesh number **(D)** were all compared between hBECs and patients-derived ECFCs cultured in 25% GM and 25% GM substituted with MSCs. Representative images of the patient tubes at 3 h demonstrate the differences in angiogenic potential between patients **(E)**. Photomicrographs also demonstrated the presence and location of MSCs (red arrows) in culture with patient-derived ECFCs. GM, growth media, MSC, mesenchymal stem cell. Statistics: **(Ai)** Tube length in 100% GM: #*p* < 0.05 100% GM vs. patient 3, patient 5, patient 6; *All groups < 0.05 except for patient 3 vs. patient 5; ∧*p* < 0.05 100% GM vs. patient 5 and patient 6, patient 3 vs. patient 5 and patient 6. **(Aii)** Tube length in 25% GM: #*p* < 0.05 25% GM vs. patient 3 and patient 6; patient 3 vs. patient 5; patient 3 vs. patient 6; *All groups *p* < 0.05 except for 25% GM vs. patient 5 (ns); ∧*p* < 0.05 25% GM vs. patient 3; patient 3 vs. patient 5. **(Aiii)** Tube length in 25% GM + MSC: #*p* < 0.05 25% GM + MSC vs. patient 3 + MSC and patient 5 + MSC; ∧*p* < 0.05 25% GM +MSC vs. patient 6 + MSC. **(Bi)** Junctions: 25% GM: #*p* < 0.05 25% GM vs. patient 6; *p < 0.05 25% GM vs. patient 3; patient 3 vs. patient 5; patient 3 vs. patient 6; ∧*p* < 0.05 25% GM vs. patient 3; patient 3 vs. patient 5. **(Bii)** Junctions 25% GM + MSC: *p < 0.05 25% GM + MSCs vs. patient 5 + MSC; ∧*p* < 0.05 25% GM + MSCs vs. patient 3 + MSC; patient 3 + MSC vs. patient 5 + MSC; patient 3 + MSC vs. patient 6 + MSC. **(Ci)** Branch number 25%GM: #*p* < 0.05 25% GM vs. patient 3 and patient 6; patient 3 vs. patient 5; *p < 0.05 25% GM vs. patient 3; patient 3 vs. patient 5; patient 3 vs. patient 6; ∧*p* < 0.05 25% GM vs. patient 3; patient 3 vs. patient 5. **(Cii)** Branch number 25% GM + MSC: #*p* < 0.05 25% GM+MSC vs. patient 3 and patient 5 + MSC: *p < 0.05 25% GM+MSC vs. patient 3 + MSC; ∧*p* < 0.05 patient 1+MSC vs. patient 6 + MSC. **(Di)** Mesh number in 25% GM: ∧*p* < 0.05 25% GM vs. patient 3; patient 3 vs. patient 5; patient 3 vs. patient 6. **(Dii)** Mesh number 25% GM + MSC: not significant.

Patient derived ECFCs were also challenged in tube forming assays at different growth medium concentrations with or without the addition of MSCs and compared to hBECs (Figure 7A). Time-lapse microscopy revealed the ability of all patient-derived cells to form tubes over a 6 h period yet the rate of formation differs between hBECs and each patient. Lowering the concentration medium from 100% to 25% seemed to have the greatest effect on hBECs and reduced the total length of tubes (Figures 7Ai,ii). Substituting the growth medium with MSCs also had little effect on total tube length (Figure 7Aiii). Patient 3 had significantly more junctions, branches and meshes than other patients and hBECs regardless of the presence of MSCs in the growth medium (Figures 7B–D). MSCs can be observed in the patient-derived assays at the base of tubes acting as anchors, rather than branching themselves (Figure 7E).

## Discussion

This study provides an unbiased, imaged-based method to determine the optimal timing of ECFC passaging, when isolated from small volumes of blood from ESRD patients. We furthermore recommend the culture conditions for consistent tube forming assays utilizing continuous live cell imaging combined with time-lapse microscopy. Subsequent analysis of 20 angiogenic parameters was conducted in an unbiased and reproducible way using image analysis software FIJI, an open source image processing package.

The starting volume of blood is an important parameter to report, as the number of circulating progenitors will increase when larger volumes of blood are obtained. Previous studies have successfully isolated ECFCs using volumes of blood ranging from 15 to 40 mL and from a variety of sources, including cord blood (Mead et al., 2008; Chevalier et al., 2014), from healthy controls (Gulati et al., 2003; Mukai et al., 2008; Minami et al., 2015), or directly from patients groups (Krieter et al., 2010; Brittan et al., 2013). Some studies investigating ECFC formation do not report their blood volumes (Gulati et al., 2003; Minami et al., 2015), which limits the ability for results to be reproduced. The frequency of ECFCs in the peripheral circulation occurs about 1 in 20 mL of blood (Ingram, 2004). Therefore, it has been suggested that ~80–100 mL of blood is required to obtain proliferative colonies, which is not viable from patients with diseases such as in ESRD.

ECFCs are identified in culture according to their cobblestone morphology, high proliferative capacity and ability to be passaged. Optimal ECFC passaging after initial culture from the PBMC fraction of blood has previously been reported as day 14 (Prasain et al., 2012). In this study however, we found that ECFCs from ESRD patients started to appear as late as day 17. We therefore propose that rather than a specific time point be used to determine passage time, colony cell number as determined by image analysis can be used to quantitatively and accurately passage cells. ECFC appearance is generally visualized daily by microscopy which involves the removal of cells from the incubator for extended periods. With longer durations, the cells undergo temperature changes that can modify their growth kinetics (Francescone et al., 2011). Therefore, the use of a customized live cell imagining microscope, equipped with a stage-top incubator, temperature control and a CO_2_ humidifier, eliminates time pressure from these experiments, especially during investigations of numerous plates. Additionally, the ability to identify the same colony over sequential days was enabled by using the “Mark and Find” function of the LX microscope (Leica) with subsequent analysis of colony size easily determined using the macro described.

Here we show that the timing of ECFC appearance in culture is widely varied in patient-derived cells. Based on our findings that blood pressure was the best predictor of ECFC formation (Huuskes et al., 2018) it was hypothesized that blood pressure would also influence the kinetics of ECFC in culture. However, there was no correlation between patient systolic or diastolic blood pressures and the time it took for ECFCs to first appear or mature in culture. Since it has been suggested that uremic toxins can influence EPC function (Krieter et al., 2010; Lapidos et al., 2014), we next investigated if the patients age or time on dialysis affected ECFCs in culture. Again, there was no correlation between patient clinical characteristics and ECFC culture. Confounding factors such as patients last dialysis session and even dietary habits (Mena et al., 2018) may have influenced our ability to tease apart the reasons for such different ECFC kinetics *in vitro*.

A limitation of this study is the inability to perform these angiogenic assays on healthy controls. We observed that patient-derived ECFCs had a forming efficiency of 50%, which is similar to the emergence of ECFC colonies that Gu et al. observed when they compared healthy controls (40%) to patients with renal cell carcinoma (87.8%) (Gu et al., 2015). Likewise, Zhao and colleagues determined that ECFC appear in culture and proliferate significantly more when cultured from cells obtained from patients with ESRD, compared to healthy controls. Additionally, patient-derived ECFCs perform in a similar way to healthy control-derived ECFCs in regard to the ability to form tubes on Matrigel; migrate through a transwell; and had similar cytokine expression profiles (Zhao et al., 2014). We were also unable to determine if the time taken for ECFCs to mature over time of culture correlated with ESRD. There are limited studies that report the time taken to obtain ECFC in both healthy and disease states, however, Minami and colleagues classified the angiogenic potential of ECFCs based on their time of emergence in culture. They determined that “late”-outgrowth EPCs (LOC) isolated from healthy volunteers appeared in culture, as determined by a change of cell morphology to a cobble stone appearance, from 17 to 23 days. The LOCs performed significantly better during *in vitro* angiogenic assays promoted reperfusion in a hind-limb ischemia model of vascular injury (Minami et al., 2015). The ECFCs that were subcultured and challenged in tube formation assays in this study were derived from patient cells that first had a change in morphology from 10 to 15 days. It is also interesting to note that no ECFCs emerged from patient cells after days 17, which could indicate that ESRD is contributing to an impaired angiogenic response.

Measuring and quantifying the angiogenic potential of cells utilizing *in vitro* assays such as tube formation is important for understanding endothelial cell function and mediators of angiogenesis. Currently, there is no consensus or standardization for performing tube forming assays, therefore the optimization of tube forming assays will ensure accurate and reproducible results. By utilizing a microscope that replicates an incubator, with controlled temperature, pH, and humidity, in conjunction with the ability to revisit multiple points and automatically capture images, time-lapse microscopy can be a powerful tool to analyze data at multiple time-points over an extended period of time for numerous assays, including tube formation (Jonkman et al., 2014).

Current reports show the method of performing tube forming assays varies according to plate size, cell number, time of assay and end-point determinants (Choi et al., 2004; Zhao et al., 2014; Gu et al., 2015). Here, we provided evidence to demonstrate that a cell number >0.5 × 10^4^ cells in a 96 well-plate resulted in tube formation that could not be quantified in a consistent manner using the angiogenesis plugin for FIJI. Furthermore, comparison of tube formation at 1 and 6 h demonstrated a significant decrease in all parameters measured, indicating that these assays decline as early as 6 h, which is earlier than the reported 24 h where significant apoptosis occurs disrupting the tubes (Francescone et al., 2011).

Most studies that investigate the tube formation ability of endothelial cells reference one time point, usually over extended periods of 12 h (Gulati et al., 2003; Choi et al., 2004; Mead et al., 2008; Zhao et al., 2014; Minami et al., 2015). Conversely, time-lapse microscopy offers the advantage of visualizing tube formation over multiple time points and therefore fixation of tubes at specific time points (Mukai et al., 2008) is not necessary. Our findings suggest that starting cell number and concentration of GM are important for determining the success of tube formation, and the modulation of angiogenic potential with the addition of agents. Moreover, tubes formed with hBECs were observed to retract after 7 h of continuous culture, as confirmed by measurement of total tube length. This indicates time-sensitive analysis of which 12 h may be too long to fully appreciate that angiogenic potential of endothelial cells or modulators of angiogenesis. However, the use of a temperature controlled stage and CO_2_ incubator, overcomes these limitations. Furthermore the use of the angiogenesis analyzer plug-in available for FIJI (Carpentier et al., 2012; Schindelin et al., 2012) allows a fast, reproducible method to analyze over 20 angiogenic parameters. The addition of a macro programmed with the macro language of FIJI, makes this process more efficient by quickly identifying and saving images at specific time points, which can be manipulated according to the time point of interest. The use of a 96 well-plate has advantages including, minimal use of Matrigel (60 μL compared to 120 μL in bigger plates), multiple samples and variables can be imaged on the one plate and also that the majority well can be imaged using a 5x objective on the LX AF6000. Positioning the objective within the well was additionally deemed important where cells in the center of the well are easier to identify based on shadows that form.

We were able to show that hBECs and patient-derived ECFCs were able to undergo tube formation, and, contrary to our hypothesis that MSCs would increase the angiogenic potential of cultured cells, MSCs contributed little to angiogenesis. It was noted, however, that MSCs act as an anchor, rather than contribute directly to total tube length, and sit at junction points over the course of the tube forming experiment. This is surprising as the angiogenic properties of MSCs and MSC-derived exosomes are well-documented (Gong et al., 2017; Gonzalez-King et al., 2017), however future investigations should look at isolating and delivering exosomes to patient derived ECFCs to determine their full impact on angiogenesis.

EPCs and ECFCs may hold promise as a therapy for vascular disease, however, the optimal culture conditions and measurement of angiogenic potential of these cells *ex vivo* is not fully elucidated. In summary, we describe analysis macros, in conjunction with time-lapse microscopy as a useful tool that can be used to correctly identify ECFCs ready for passaging to increase culture success. Additionally, we provide evidence that cell seeding densities and media concentration and length of assay are important considerations when performing tube assays on Matrigel. Further, we demonstrate that these culture conditions can be used to demonstrate tube formation in patient derived cells.

## Ethics Statement

This study was carried out in accordance with the recommendations of Monash University Human Research Ethics Committee with written informed consent from all subjects. All subjects gave written informed consent in accordance with the Declaration of Helsinki. The protocol was approved by the Monash University Human Research Ethics Committee, Project number CF16/402 – 2016000182.

## Author Contributions

SR and CS contributed to intellectual experimental design and funding. BH performed the research and analyzed the data. RD performed and analyzed flow cytometry data. PK coordinated patient sample collection and ethical approval. All authors prepared the final manuscript.

### Conflict of Interest Statement

The authors declare that the research was conducted in the absence of any commercial or financial relationships that could be construed as a potential conflict of interest.
