# Supplementary material for: The Use of Live Cell Imaging and Automated Image Analysis to Assist With Determining Optimal Parameters for Angiogenic Assay in vitro
Source: Front Cell Dev Biol. 2019 Apr 10;7:45. doi: 10.3389/fcell.2019.00045 (PMC6468051; doi:10.3389/fcell.2019.00045)
Supplement: Supplementary file 7 [file Data_Sheet_1.pdf]

Supplementary Material

The use of live cell imaging and automated image analysis to assist with determining optimal parameters for angiogenic assay in vitro.

Brooke M Huuskes[1*], Ryan J Debuque[2], Peter G Kerr[3], Chrishan S Samuel[4], Sharon D Ricardo[1]

*** Correspondence:** Dr Brooke Huuskes b.huuskes@latrobe.edu.au

Additional Files 1: Supplementary figures:

Supplementary figure 1: LX AF6000 Microscope components

The Leica LX AF6000 microscope contains a custom build perspex chamber (**A**) that encapsulates the scope. Carbon dioxide is pumped from a cylinder (**B**) into a multifunction controller (**C**) which allowed the temperature and CO_2 level to be set and gives control over the flow rate. The perspex chamber was heated by two fan-forced heaters (**D**) and the CO_2 was bubbled through a reservoir containing milliQ water, providing humidity. The reservoir was connected to a custom built perspex lid (**E**) that covers any standard plate, and was held in place by screws to the heated stage. This set up allows live cells to be imaged continuously for extended periods of time under experimental culture conditions of 37˚C and 5% CO_2.

Supplementary figure 2: Software set up for optimal live cell time-lapse imaging

For quality, reproducible results, the microscope software should be set up consistently for every experiment, even when conducted on separate days. (**A**) We report that a minimal exposure time (of less than 10ms) with a low gain (below 5) is optimal for obtaining images that are suitable for analysis with the angiogenesis plug-in. The light intensity is also important for consistent images and can be adjusted if volumes of Matrigel or media used in experiments is altered. We found that an exposure of 4ms, Gain of 1 and intensity of 5 was optimal for the 5x objective. A low objective should also be used when imaging tube formation on a 96 well plate as the centre of the well is easily visible and this objective additionally captures the majority of the well.

When a time-lapse series is required to be captured, the **t** can be pushed (**B**) which will bring up the dialog box (**C**). It is important to make note of the parameters once they are set as this will determine which photos will be used in the analysis post-experiment. Here, two different time intervals were used to capture the time-lapse series, 2 minutes and 30 seconds for 6 hours, resulting in 144 images (**Ci**) or 5 minutes intervals for 7 hours resulting in 84 images (**Cii**).

The 'Mark and Find' function on the software can be found by clicking on the three squares shown in

(**B**). The experiment demonstrated (**D**) is one experiment performed in triplicate on a 96 well plate. The mark and find function allows desired wells to be imaged with high accuracy over an extended period of time however, it should be noted that focusing each channel is critical. The authors did not to use the autofocus function of this microscope (the down pointed arrow depicted in **B**) as air bubbles in the matigel or the media interfere with the autofocus function. All tube data presented in this paper were from time lapse images that have been manually focused. It is important to un check the “same stack for all” button before proceeding with focusing individual wells. Care should be taken to ensure that no air bubbles are present in either the matrigel or medium in these assays, as this will affect the images obtained and therefore the analysis.

Supplementary figure 3: Growth media concentration affects the ability of hBECs to undergo tube formation

GM was titrated to determine the correct concentration of medium that allows for the determination of an increase or decrease in tube formation ability. Tubes were cultured in 100% growth medium, or medium substituted with basal medium containing no growth factors so that a final concentration of 75%, 50% 25% 10% and 1% growth medium was obtained. Tubes were analyzed over 6 hours using time-lapse microscopy and images from each hour of the experiment are represented. Images for this figure were extracted from the time-lapse series of images with the use of the macro described in supplementary figure 4.

Abbreviations: GM, growth media

Supplementary figure 4: Tube angiogenic parameters analysed at additional time points over the 6 hour tube forming experiment on Matrigel

Total tube length and the number of junctions, branches and meshes were analysed every hour over the 6 hours as described in figure 3. The remainder of the time points analysed are shown here at 2, 4 and 5 hours.

Additional Files 2: Videos of tube formation

Time-lapse images of tube formation over 6 hours were opened in FIJI and exported as an AVI. This allows the kinetics of tube formation to be visualized over the 6 hour period and gives an indication of the correct cell number that should be used. A cell density of 2-10x10^4 (Video 3-6) cells is too great for proper tube formation and results is holes being formed within the cells, rather than the cells reaching for each other to anastomose. Similarly, cell densities of 0.2x10^4 (Video 1) are insufficient for tube formation whereas 0.5x10^4 is optimal (Video 2) Using this the rate of tube formation over time can also be investigated.

frontiers
