# Supplementary figures and images for: The Use of Live Cell Imaging and Automated Image Analysis to Assist With Determining Optimal Parameters for Angiogenic Assay in vitro
Source: Front Cell Dev Biol. 2019 Apr 10;7:45. doi: 10.3389/fcell.2019.00045 (PMC6468051; doi:10.3389/fcell.2019.00045)

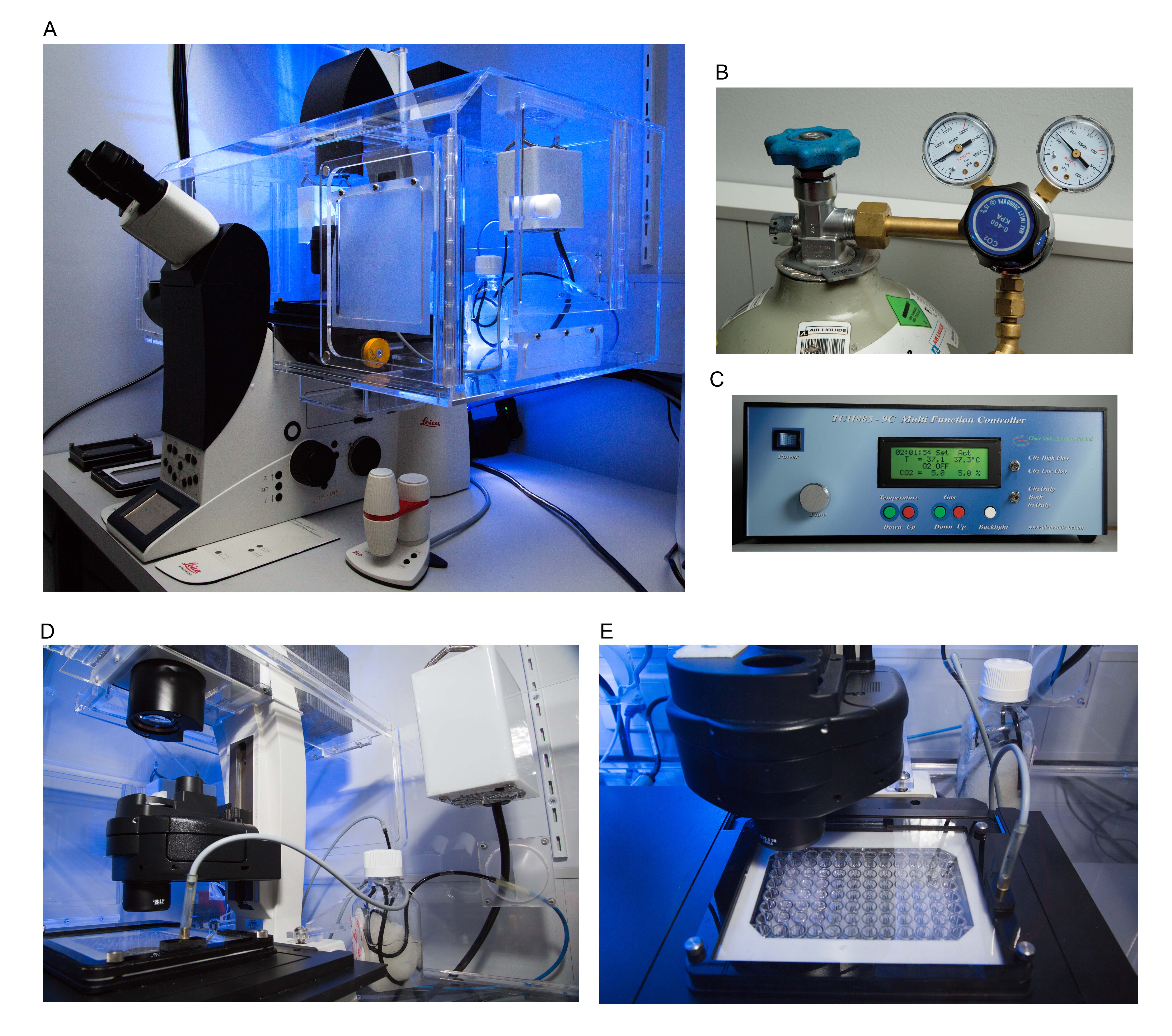

Supplement: Supplementary file 8 [file Image_1.JPEG]

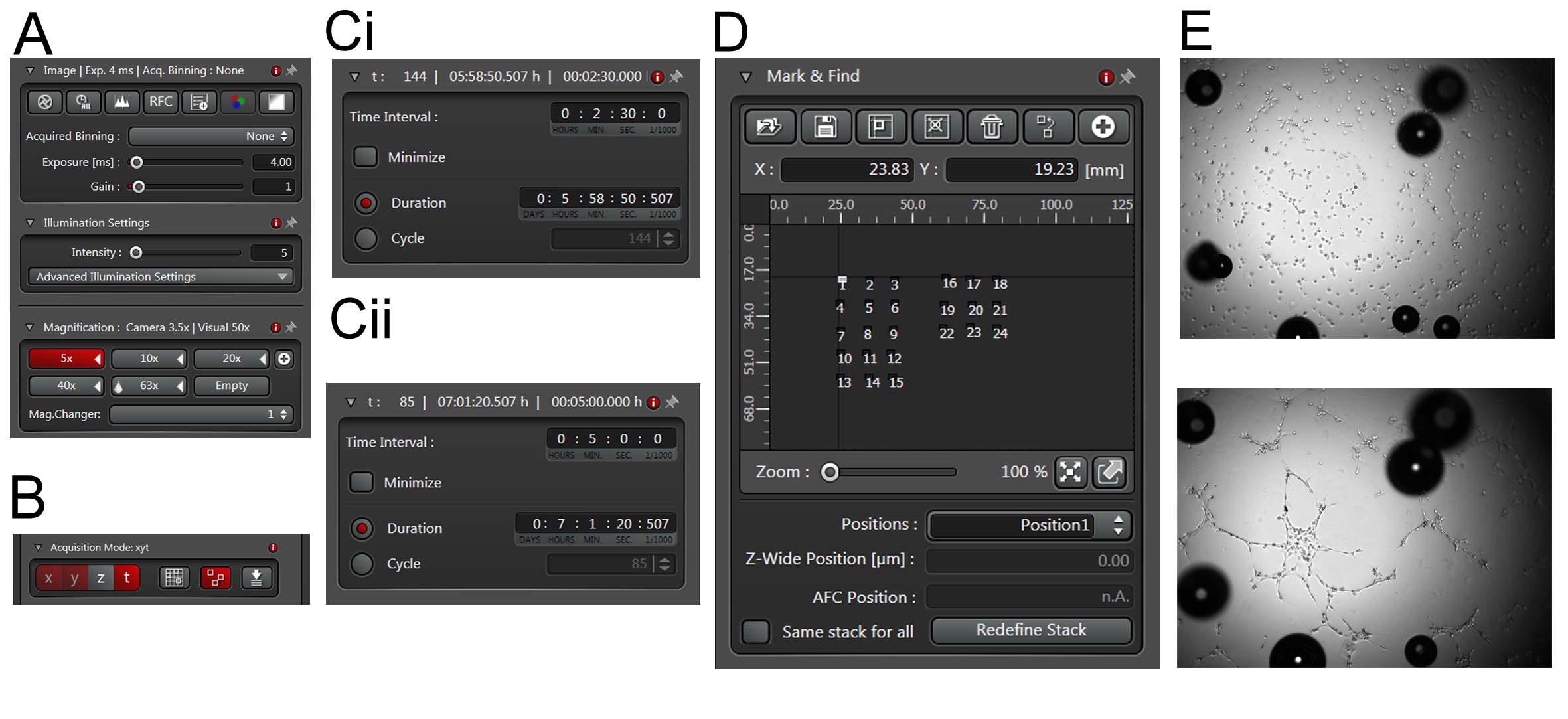

Supplement: Supplementary file 9 [file Image_2.JPEG]

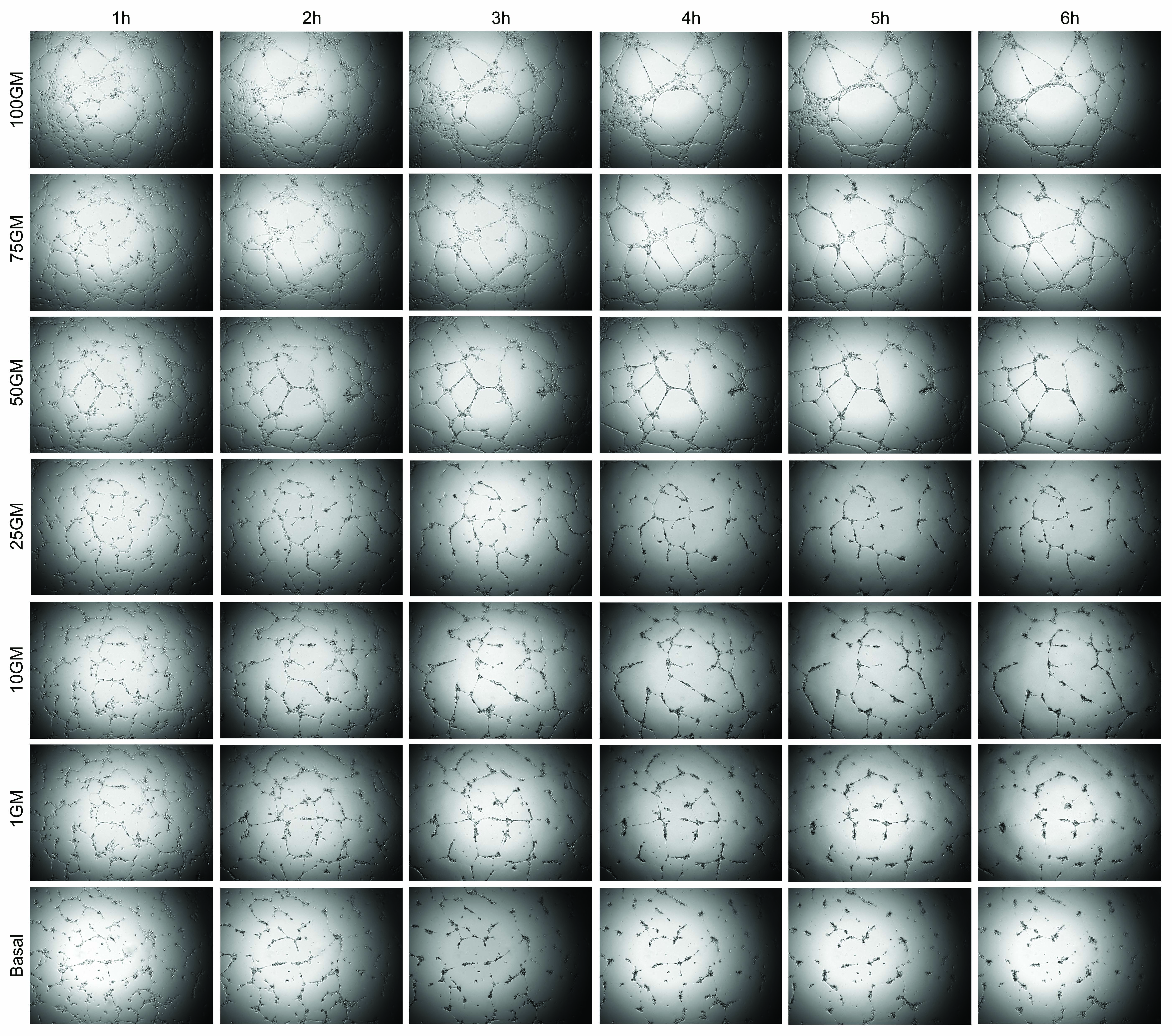

Supplement: Supplementary file 10 [file Image_3.JPEG]

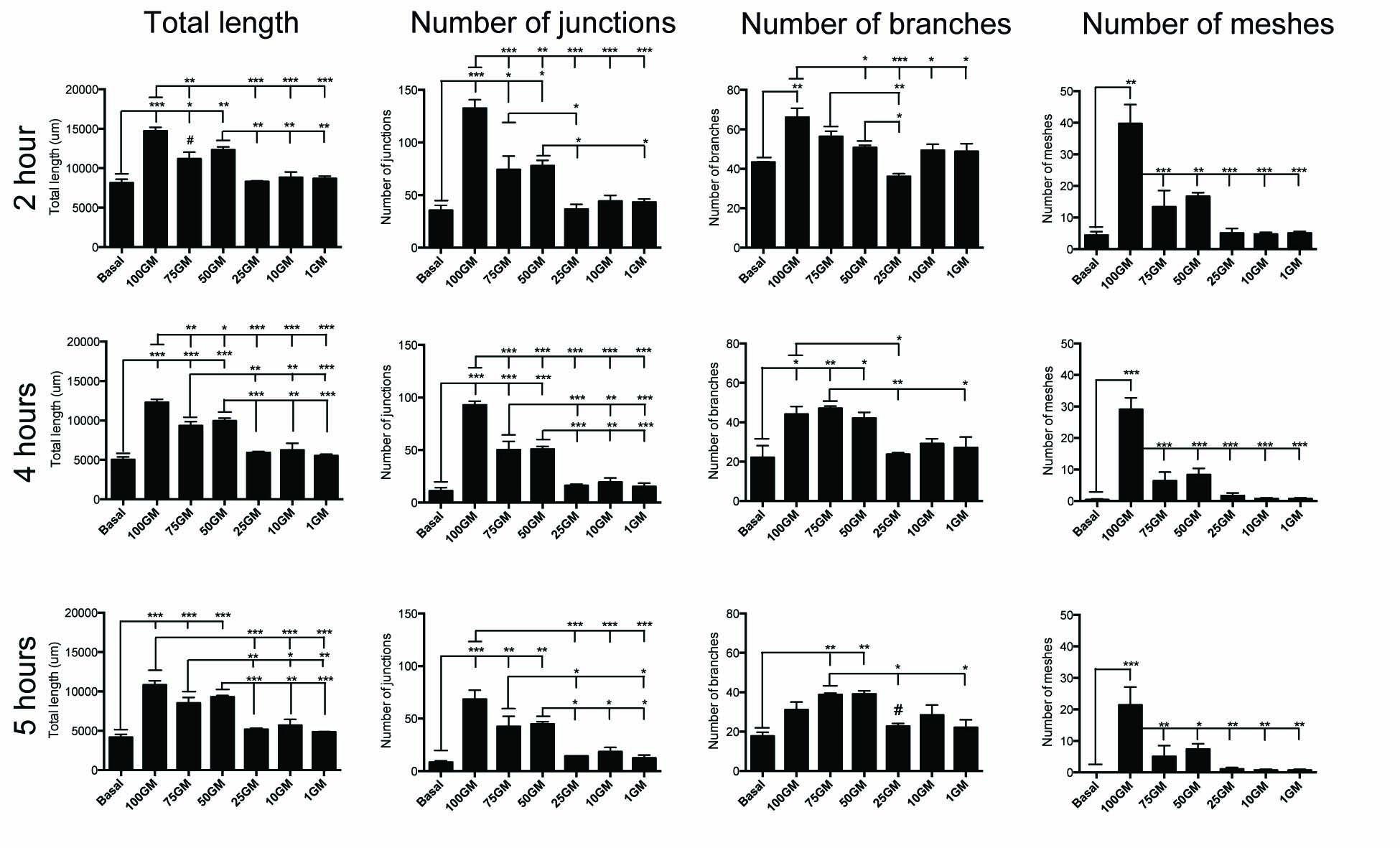

Supplement: Supplementary file 11 [file Image_4.JPEG]
